# Estimated costs of the treatment of diabetic foot ulcers in a tertiary hospital in Turkey

**DOI:** 10.12669/pjms.305.5182

**Published:** 2014

**Authors:** Sakir Ozgur Keskek, Sinan Kirim, Necmettin Yanmaz

**Affiliations:** 1 Sakir Ozgur Keskek, Department of Internal Medicine, Numune Training and Research Hospital, Adana, Turkey.; 2 Sinan Kirim, Department of Internal Medicine, Numune Training and Research Hospital, Adana, Turkey.; 3 Necmettin Yanmaz, Department of Internal Medicine, Numune Training and Research Hospital, Adana, Turkey.

**Keywords:** Diabetic foot ulcers, Cost of treatment, Type 2 diabetes mellitus

## Abstract

***Objective:*** The prevalence of diabetes and its related complications are increasing, and a considerable portion of healthcare expenditures is spent worldwide on diabetes and its complications. In this study, we investigated the estimated treatment costs of diabetic foot ulcers in a tertiary hospital in Turkey.

***Methods:*** A total of 203 patients with type 2 diabetes mellitus were included in this retrospective study. The study group comprised 91 patients with foot ulcers and the control group comprised 112 patients without any chronic complications. Their demographic characteristics, HbA1c levels and the length of hospital stay were recorded. The patients’ bills, received from the hospital billing departments, were analysed.

***Results:*** The average cost of diabetes patients with foot ulcers per person was calculated as 976.1±253.6 USD while it was 430.3±144.2 USD for diabetes patients without any chronic complications; thus, there was a significant difference between the average cost of these groups (p<0.001). Similarly, there were significant differences between the groups according to the costs of drugs, equipment and services (p<0.001).

***Conclusion:*** The estimated cost of treatment of diabetic foot ulcers is high in Turkey. It will continue to be a heavy economic burden if preventive measures are not taken.

## INTRODUCTION

Diabetes mellitus (DM) imposes a high economic burden on individuals and societies. Furthermore, in the case of co-morbidities and/or complications, the treatment costs are rising. Macrovascular and microvascular complications are the most significance factors of this disease that lead to high treatment costs and mortality.^1^^-^^3^

One of the major morbidities and a significant macrovascular complication of diabetes is the diabetic foot ulcer. It is also one of the causes of hospital admissions for people with DM in developed countries.^4^ The prevalence of foot ulceration due to diabetes is 6% in the US. Moreover, this percentage is higher in developing countries.^5^^,^^6^ Problems associated with diabetic foot ulcers are common throughout the world, resulting in major economic consequences for the patients, their families and societies.^7^ Diabetic foot ulcers cause a high economic burden due to the long term treatments, long hospital stay and loss of labour.

In Turkey, the estimated prevalence of DM is 14.8% according to the 2013 IDF atlas.^2^ Nevertheless, there is not enough information about the prevalence and the treatment costs of diabetic foot ulcers in our country. In this study, we investigated the estimated costs of diabetic foot ulcers in a tertiary hospital in Turkey.

## METHODS

In this study, data from 203 patients with diabetes mellitus who were treated at the Internal Medicine Clinic of Adana Numune Training and Research Hospital from 2010 to 2012 were studied retrospectively. The institutional review board of our hospital approved the study. Patients with a diagnosis of gestational diabetes, malignancies, patients with diabetes who were hospitalised because of diseases other than diabetes and patients with diabetes who were hospitalised because of complications other than diabetic foot ulcers (except acute complications) were excluded. Therefore, we did not include patients with diabetic retinopathy, nephropathy, coronary heart disease, neuropathy and infectious diseases.

Patients were divided into two groups. The study group consisted of 91 patients with diabetic foot ulcers and the control group consisted of 112 patients with diabetes without any chronic complications or co-morbidity. Patients in the control group were hospitalised due to hyperglycaemia. The length of the hospital stay and the HbA1c levels of all patients were recorded. At our institute, the Tosoh G7 (Belgium) model analyser was used for analysing the HbA1c levels by HPLC. HbA1c percentages (NGSP) were converted to IFCC (mmol/mol) with 10.93×(NGSP%)-23,5 formula. Invoices of all patients were obtained from the billing department. The costs of patients were analysed from hospital charges to third-party payers. The average cost of treatment was calculated based on the sum of the costs of services, drugs and equipment. The costs calculated in this study were generalised costs since they are standardised by third-party payers and the Turkish government.

The drugs used comprised oral anti-diabetics, insulin preparations, antibiotics, intravenous fluids, antihypertensive drugs and anti-hyperlipidemia drugs. The equipment used included intravenous catheters, syringes, blood glucose monitoring strips, bandage supplies and foley catheters. The cost of services consisted of hospitalisation fees, venous access, intramuscular injection, laboratory services, electrocardiography, radiological imaging, oxygen therapy, wound debridement and surgical operations. Each patient’s cost of treatment was calculated from a single hospital stay. All costs in this study were calculated in Turkish Liras (TL) and converted to United States Dollars (USD). The year and conversion rate were 2013 and 0.5, respectively (1 USD=2TL).

The MedCalc 12.7 software program (MedCalc, Belgium) was used for the statistical analysis, and data were reported as the mean ± standard deviation (sd). Chi square and Kolmogorov-Smirnov tests were used to compare the categorical measurements (gender distribution) between the groups, and to show the normal distribution of the quantitative measurements, respectively. The Student T test or Mann Whitney U test was used for the comparison of the cost of drugs, equipment and services among the two groups. A correlation coefficient was used to analyse the degree of association between HbA1c, the length of the hospital stay and the costs (Pearson correlation coefficient (r), with P-value and 95% CI for r). A log transformation was used for the variables that were not normally distributed. The probability of making a Type I error (alpha, significance) is 0.05.

## RESULTS

The mean ages of the groups were 57.6±6.2 and 54.1±11.9 years old, respectively. There were 38 (41.8%) women and 53 (58.2%) men in the study group, and 66 (58.9%) women and 46 (41.1%) men in the control group. There were statistical differences in the ages and gender distributions of the two groups (p=0.04 and 0.02, respectively; Table 1). Patients with diabetic foot ulcers were older than patients without diabetic foot ulcers. The majority of the study group were males while the majority of the control group were females. HbA1c levels and the length of hospital stay were found to be 10.1±1.6 (86.8±17.4 mmol/mol) and 14.0±5.0 days, respectively, in patients with diabetic foot ulcers while they were 9.2±1.4 (77.0±15.3 mmol/mol) and 5.9±2.1 days, respectively, in the control group. There were statistically significant differences between the groups (p<0.001, respectively; Table-I).

The costs of drugs and equipment, the costs of services and average costs were 415.5±131.2 USD, 560.6±212.6 USD and 976.1±253.6 USD, respectively, in the study group, while they were 157.3±63.0 USD, 273.0±106.4 USD and 430.3±144.2 USD, respectively, in the control group. There were statistically significant differences between the groups according to the types of costs (p<0.001, respectively; Table-I). 

In the study group, the men were younger than the women and the HbA1c levels of the men were higher than those of the women (p=0.01 and p=0.04, respectively). There was no statistical difference between men and women according to the length of hospital stay (p=0.72; Table-II).

The differences between the male and female patients according to the costs of drugs and equipment, services and the average costs were not statistically significant in the study group (p=0.49, 0.23 and 0.27, respectively; Table-II). 

As expected, the average cost was positively correlated with the length of the hospital stay (p=0.002, r=0.319). The cost of drugs and equipment was positively correlated with the HbA1c percentage and the length of the hospital stay (p=0.006, r=0.281; p<0.001, r=0.603; respectively). The length of the hospital stay also correlated with the HbA1c percentage (p<0.001, r=0.44; Table-III).

## DISCUSSION

In this study, we investigated the estimated treatment costs of diabetes patients with foot ulcers. We have shown that the estimated costs of these patients were higher than those for hyperglycaemic patients without any chronic complications. The average cost, the cost of drugs and equipment were positively correlated with the length of the hospital stay. Moreover, the cost of drugs and equipment and the length of the hospital stay were positively correlated with HbA1c.

**Table-I:** The treatment costs and properties of the study and control groups.

	***Study group*** ***N=91***	***Control group*** ***N=112***	***p***
Age	57.6±6.2	54.1±11.9	0.04
Female N (%)	38 (41.8%)	66 (58.9%)	0.02
The length of hospital stay (days)	14.0±5.0	5.9±2.1	<0.001
HbA1c % (mmol/mol)	10.1±1.6 (86.8±17.4)	9.2±1.4 (77.0±15.3)	<0.001
Cost of drugs and equipments (USD)	415.5±131.2	157.3±63.0	<0.001
Cost of services (USD)	560.6±212.6	273.0±106.4	<0.001
Average cost (USD)	976.1±253.6	430.3±144.2	<0.001

**Table-II:** Properties of patients with diabetic foot ulcers according to the gender

***Patients with diabetic foot ulcers***	***Men*** ***N=53***	***Women*** ***N= 38***	***p***
Age	56.2±6.5	59.6±5.3	0.01
HbA1c % (mmol/mol)	10.4±1.7 (86.8±17.4)	9.7±1.2 (77.0±15.3)	0.04
The length of hospital stay (days)	13.8±4.7	14.3±5.4	0.72
Cost of drugs and equipments (USD)	423.4±138.9	404.4±120.5	0.49
Cost of services (USD)	577.3±196.1	537.3±234.3	0.23
Average cost (USD)	1000.8±233.7	941.8±278.6	0.27

**Table-III:** Correlation analyses of variables

	***The length of hospital stay ***	***HbA1c ***
Average cost (USD)	r=0.319 p=0.002	r=0.06 p=0.524
Cost of drugs and equipments (USD)	r=0.60 p<0.001	r=0.281 p=0.006
Cost of services (USD)	r=0.03 p=0.976	r=-0.09 p=0.380
The length of hospital stay		r=0.443 p<0.001

 Previous studies have shown that high costs of treatment were largely due to the diabetes mellitus related complications. In the study by Al-Maskari et al., the direct medical cost of diabetes in the United Arab Emirates was reported to be 1605 USD. This cost increased 6.4 times for patients with macrovascular complications.^8^ Kim et al. reported that the annual direct medical costs of patients with only macrovascular, only microvascular or both macrovascular and microvascular complications were 2.7, 1.5 and 2.0 times higher, respectively, than those of patients without complications.^9^

 High treatment costs are caused by surgical interventions in addition to the medical treatments. In Prompers et al.’s study, they determined that the direct cost of a diabetic foot ulcer was 3771 Euros, and in the case of extremity amputation, the total cost was 16414 Euros.^10^ Similarly, Ali et al. conducted a retrospective study on 383 diabetes patients in Pakistan. They reported high costs and economic burdens associated with diabetic foot disease.^11^ In the study by Rezende et al., the high social and economic impact of diabetic foot disease in Brazil (a developing country) was reported.^12^

We also showed high treatment costs of chronic complications related to diabetes in our previous study.^3^ The treatment costs of neuropathy and peripheral artery disease were significantly higher in that study. The result of the current study is supported by our previous research since atherosclerosis and neuropathy play a significant role in the development of diabetic wounds.^13^

Hyperglycaemia can be found at any stage of the disease, while foot ulcers can develop after neuropathy and atherosclerosis due to prolonged hyperglycaemia. For this reason, increased age may be expected for diabetic foot ulcer patients compared to hyperglycaemic patients. Further reinforcing this link, patients with diabetic foot ulcers were older than patients with hyperglycaemia in the current study. Additionally, a higher percentage of HbA1c in patients with foot ulcers was probably caused by prolonged uncontrolled hyperglycaemia.

The length of the hospital stay is an important factor which leads to the high costs. According to Lee et al.’s study, the length of the hospital stay is a measure of hospital activity, healthcare utilisation and a determinant of hospitalisation costs.^14^ In accordance with this link; we have found longer hospital stay durations in patients with foot ulcers in our study. Patients with diabetic foot ulcers may need to stay longer because of uncontrolled hyperglycaemia, long-term wound care, infections, debridements, amputations and other newly developed complications. All these factors increase the treatment costs. 

In this study, the majority of the patients in the study group were men while the majority of the patients in the control group were women. The high prevalence of foot ulcers in men might be due to different working conditions, negligence and lower levels of hygiene in our study population.

This study had some limitations. First, it would have been beneficial if we had separated diabetic foot ulcers according to the severity of the disease or the treatment methods (medical or surgical). Second, annual costs can be more useful in devising appropriate plans than costs incurred during a single hospital stay. However, this is the first study in our country, and we believe that the data presented here may provide useful knowledge about the estimated direct medical costs of diabetic foot ulcers in Turkey. 

In conclusion, the economic burden of diabetic foot ulcers on individuals and society is high in our region. The treatment of this health problem is costly due to the high costs of the longer hospital stay, drugs, equipment and hospital services. To reduce the cost of DM and its complications treatment, not only health professionals but also patients and their relatives must contribute toward DM management. Health administrators should increase the number of experienced staff dealing with DM and make health services easily accessible. Management of DM-related complications should be developed and promoted. Additionally, education should be continuously provided to the patients. Finally, further studies from our country are needed for to show the treatment cost of diabetic foot ulcers more clearly.

## Authors Contribution:

**SOK** conceived, designed and did statistical analysis & editing of manuscript.

**SOK, SK & NY** did data collection and manuscript writing.

**SOK** takes the responsibility and is accountable for all aspects of the work in ensuring that questions related to the accuracy or integrity of any part of the work are appropriately investigated and resolved.

## Notes

***Funding: ***None

## Notes

**IRB approval date and number:** ANEAH. EK.2013-6, 8.01.2013
